# Comparative Goniometric Analysis of Tibial Torsion and Q-angle Between Sedentary Young Adults and Amateur Athletes: A Cross-Sectional Comparative Study With Prospective Data Collection

**DOI:** 10.7759/cureus.110634

**Published:** 2026-06-10

**Authors:** Kumari Ayushi, PrajnaParamita Samanta, Mamata Panigrahi, Nihar Ranjan Mohanty, Priyabrata Dash

**Affiliations:** 1 Anatomy, Kalinga Institute of Medical Sciences, Bhubaneswar, IND; 2 Physiotherapy, KIIT School of Physiotherapy, Bhubaneswar, IND

**Keywords:** amateur athletes, goniometric measurement, lower extremity alignment, patellofemoral biomechanics, q-angle, quadriceps angle, rotational alignment, sedentary young adults, sports medicine, tibial torsion

## Abstract

Background: Lower-limb alignment parameters, particularly tibial torsion and the quadriceps angle (Q-angle), are key determinants of patellofemoral mechanics. Their comparative profiles across different activity levels in young adults remain inadequately characterized. The 18-25-year age window was chosen because lower-limb skeletal alignment is mature by 18 years, while age-related degenerative knee changes are uncommon below 25 years, minimizing both developmental and degenerative confounding.

Objective: To compare tibial torsion and Q-angle between sedentary young adults and amateur athletes aged 18-25 years and determine the influence of habitual physical activity on these lower extremity rotational alignment parameters.

Methods: A cross-sectional study with prospective data collection was conducted at the Department of Anatomy, KIMS, in collaboration with the KIIT School of Physiotherapy, Bhubaneswar, between February 2024 and January 2026 after Institutional Ethics Committee approval (KIIT/KIMS/IEC/1510/2024). A total of 400 participants (200 sedentary, 200 amateur athletes; 100 males and 100 females per group) were recruited by convenience sampling with frequency matching of age and sex. Sedentary participants were operationally defined as performing less than 30 minutes of moderate-intensity physical activity per week and amateur athletes as performing at least three structured training sessions per week of 60 minutes or longer at a non-elite level. Q-angle and tibial torsion were measured by goniometry in the supine position by a single trained examiner; each measurement was repeated three times and averaged. Shapiro-Wilk testing demonstrated non-normal distributions, so variables are expressed as median with interquartile range (IQR), and groups were compared using the Mann-Whitney U test with rank-biserial correlation (r) as effect size; 95% CIs for medians were obtained by bias-corrected and accelerated bootstrap with 2000 resamples. Significance was set at p < 0.05.

Results: Groups were age-matched (both medians 22 years; IQR 20-24; p = 0.901). Sedentary participants had higher BMIs (median 23.81 kg/m², IQR 21.2-26.9 vs. 22.98, IQR 20.0-25.2; p < 0.001, r = 0.20). The Q-angle was higher in sedentary than sports participants (median 16°, IQR 14-18 vs. 15°, IQR 13.7-16.4; p = 0.004, r = 0.17, small effect). Tibial torsion was markedly greater in sports than sedentary participants (median 20.9°, IQR 19.0-22.7 vs. 18°, IQR 17.0-19.2; p < 0.001, r = −0.63, large effect); bootstrap 95% CIs for the medians (20.1-21.3 vs. 18.0-18.3) did not overlap, and all values remained within the physiological range of 15-30°. Total leg length did not differ significantly between groups (median 91 cm, IQR 88-95.2 vs. 90 cm, IQR 88.3-92.6; p = 0.126).

Conclusions: Regular sports participation is associated with a lower Q-angle (median difference ≈1°; small effect) and a markedly greater tibial torsion (median difference ≈3°; large effect) in young adults, reflecting activity-induced biomechanical adaptations. Elevated Q-angle in sedentary individuals may reflect higher patellofemoral loading, whereas increased tibial torsion in athletes represents adaptive rotational remodeling within physiological limits.

## Introduction

The lower limb functions as an integrated biomechanical system in which the alignment of the pelvis, femur, tibia, and foot collectively determines load transfer, postural stability, and efficiency of locomotion [1]. Among the clinical parameters used to characterize this alignment, the quadriceps angle (Q-angle) is operationally defined as the acute angle formed at the center of the patella by two lines: a proximal line drawn from the anterior superior iliac spine (ASIS) to the center of the patella and a distal line drawn from the center of the patella to the tibial tuberosity. It represents the resultant line of action of the quadriceps muscle on the patella during knee extension and is reported in degrees [2,3]. Deviations from normal Q-angle values of 10-14° in males and 13-17° in females have been consistently linked to patellofemoral pain syndrome (PFPS), anterior knee pain, chondromalacia patellae, and a spectrum of overuse injuries [4-6].

Tibial torsion represents the axial rotation of the tibia along its longitudinal axis, quantified as the angle between the proximal tibial axis at the knee and the distal bimalleolar axis at the ankle [7,8]. External tibial torsion laterally displaces the tibial tuberosity, thereby increasing the Q-angle and contributing to lateral patellar instability [8]. Because these two parameters are biomechanically related, understanding their distribution across populations with differing levels of habitual physical activity is of considerable clinical and epidemiological importance [1,9].

For the purposes of this study, the term sedentary young adults refers to individuals aged 18-25 years who do not engage in any structured sports or exercise programs and who accumulate less than 30 minutes of moderate-intensity physical activity per week, while amateur athletes refers to individuals of the same age range who participate in regular athletic or recreational sport activity, comprising at least three training sessions per week of 60 minutes or longer, but who do not compete at the elite or professional level. The 18-25-year age window was chosen because lower-limb skeletal alignment is essentially mature by 18 years of age, while age-related degenerative knee changes (such as Kellgren-Lawrence grade 1) are uncommon below 25 years; this age range therefore minimizes both developmental and degenerative confounding and corresponds to the typical age band of university-level amateur athletes.

Despite the clinical significance of Q-angle and tibial torsion, measurement challenges persist. Goniometric assessment, while clinically accessible, is subject to inter-observer variability due to soft-tissue landmark identification [3]. Although computed tomography (CT) and radiographic imaging provide superior accuracy, their cost and radiation exposure limit routine clinical use [10,11]. Goniometric measurement of Q-angle and tibial torsion has been previously validated against CT-based reference standards in direct same-subject comparisons [12], and recent systematic reviews continue to support its clinical applicability when standardized protocols are used [3,13]. Recent studies nonetheless report that inter-observer variability persists in goniometric measurement, reinforcing the need for standardized protocols and population-stratified reference data [12,13].

Beyond measurement issues, three substantive gaps exist. First, most previous studies have examined Q-angle and tibial torsion separately rather than evaluating them together as interrelated lower-limb alignment parameters. Second, available comparative data frequently focus on elite athletes or exclusively sedentary populations, leaving amateur athletes underrepresented despite their high participation in recreational and university-level sports. Third, population-specific normative values for young Indian adults remain limited, which restricts the direct applicability of international reference ranges to regional clinical and sports medicine practice [13,14]. To date, neither any published study has simultaneously compared tibial torsion and Q-angle in a population-stratified sample of young Indian adults classified by habitual activity level, nor has any study specifically characterized these parameters in the amateur athlete segment of this demographic.

This cross-sectional comparative study with prospective data collection addresses these gaps by simultaneously measuring tibial torsion and Q-angle in 400 young Indian adults, comprising 200 sedentary participants and 200 amateur athletes aged 18-25 years. The specific objectives were to compare lower-limb rotational alignment parameters between activity-defined groups, quantify the magnitude of alignment differences associated with habitual sports participation, and establish population-specific baseline values for young Indian adults. The findings may inform pre-participation biomechanical screening protocols and guide targeted injury-prevention strategies in sports medicine and rehabilitation practice [1,5,6].

## Materials and methods

A cross-sectional comparative study with prospective data collection was conducted at the Department of Anatomy, in collaboration with the KIIT School of Physiotherapy, Bhubaneswar, Odisha, India, over a two-year project period from February 2024 to January 2026, after following Institutional Ethics Committee approval on February 6, 2024 (KIIT/KIMS/IEC/1510/2024). The prospective cross-sectional comparative design was chosen as the most efficient and ethically appropriate design for establishing population-specific normative values and between-group differences in lower-limb rotational alignment. Written informed consent was obtained from participants for measurement, photography, and the use of anonymized clinical measurement images or author-created derivative schematic illustrations for academic and publication purposes. No facial features or personal identifiers are shown in the figures. All participants fulfilled the pre-defined inclusion and exclusion criteria. No participant was excluded after enrolment due to incomplete data or protocol deviation. Between-group comparisons were performed using the Mann-Whitney U test with rank-biserial correlation (r) as effect size; 95% CIs for medians were obtained by bias-corrected and accelerated bootstrap with 2000 resamples. Significance was set at p < 0.05.

Sample size

Participants were selected by convenience sampling from the student population, with frequency matching on age and sex between groups. Both groups were restricted to the 18-25-year age range, and each group was constructed to contain exactly 100 males and 100 females so that age range and sex composition were identical between groups. The a priori minimum sample size per group was computed using the standard formula for comparing the means of two independent groups [15]: n per group = 2 × (Zα/2 + Zβ)² × σ² / Δ², where Zα/2 = 1.96 corresponds to a two-tailed α of 0.05, Zβ = 0.84 corresponds to 80% statistical power, σ = 3.0° is the pooled within-group standard deviation of tibial torsion in young adults [12,16], and Δ = 1.5° is the smallest clinically meaningful between-group difference taken from the same reference studies. Tibial torsion was used as the limiting parameter; the formula yielded n = 63 participants per group. Because a pre-planned secondary analysis stratified by sex was integral to the study design, the same formula [15] was reapplied at the sex-stratum level, treating males and females within each activity group as independent comparisons in line with established guidance on subgroup sample-size calculation [17,18]. With the same σ = 3.0° and Δ = 1.5°, this yielded n = 63 per sex-stratum, i.e., 126 per activity group. To maintain a balanced 100 + 100 sex distribution within each group and to provide a margin for protocol attrition, the recruitment target was rounded upward to 200 participants per group, i.e., 400 participants in total.

Operational group definitions

Two mutually exclusive activity-defined cohorts were established.

Sedentary group: University students who did not participate in any structured sports program, physical-training regimen, or regular exercise routine, and who reported less than 30 minutes of moderate-intensity exercise per week. This cut-off corresponds to the lower bound of the World Health Organization (WHO) 2020 Guidelines on Physical Activity and Sedentary Behavior, which classify individuals failing to meet the recommended 150 minutes per week of moderate-intensity activity as “insufficiently active” [19], and is consistent with the operational definition of “sedentary lifestyle” used in earlier physical-activity epidemiology [20,21].

Amateur athlete group: Individuals engaged in regular athletic or recreational sports activities, operationally defined as participation in a minimum of three training sessions per week, each lasting at least 60 minutes, but who did not compete at a professional or elite level. This threshold matches the WHO 2020 definition of “sufficiently active” (≥150-300 minutes of moderate-intensity or 75-150 minutes of vigorous-intensity activity per week) [19] and the amateur-athlete cut-off used in comparable orthopedic and biomechanics studies [22]. These mutually exclusive definitions were applied uniformly during recruitment to ensure unambiguous classification of every enrolled participant.

Inclusion criteria

Eligible participants were apparently healthy male and female university students aged 18-25 years who were able to stand and walk independently, could undergo lower-limb anthropometric and goniometric assessment, and provided written informed consent for participation.

Exclusion criteria

Participants were excluded if they had any of the following specific conditions: (a) congenital deformity or structural abnormality affecting the lower extremities; (b) chronic systemic illness known to affect posture or gait, including diabetes mellitus, rheumatoid arthritis, or neurological disorders; or (c) lower-extremity injury within the preceding six months. As noted above, transient minor illnesses unrelated to lower-extremity biomechanics were not grounds for exclusion.

Data collection

Instruments Used

Data were collected using standardized instruments. A universal goniometer was used for angular measurements, a calibrated non-stretchable measuring tape was used for linear anthropometric measurements, a mechanical weighing scale was used for body weight measurement, and a height-measuring scale with steel tape was used for stature. A chair and wooden stool were used to support participant positioning where required, while plain white paper, tracing sheets, and a soft skin marking pencil were used for documentation of raw measurements and anatomical reference points.

Parameters Measured

Anthropometric parameters included standing height, body weight, and BMI. Standing height was measured in centimeters with the participant barefoot and in standard anatomical posture using a calibrated wall-mounted scale. Body weight was recorded in kilograms with the participant barefoot and wearing only minimal indoor clothing (light T-shirt and shorts), with pockets emptied, using a calibrated mechanical scale that was adjusted to zero before each measurement to minimize the contribution of clothing to the BMI calculation. BMI was derived in kg/m² by dividing the recorded body weight in kilograms by the square of height in meters, and the resulting values were used for between-group comparison of overall body habitus.

The primary outcome parameters were Q-angle, tibial torsion, and total leg length. Participants were instructed to wear close-fitting examination clothing during the measurement session. Before data collection, the examiner verified that the clothing allowed clear visualization and palpation of the ASIS, patella, tibial tuberosity, femoral epicondyles, and malleolar region. This step was included to reduce measurement error caused by loose or overlapping garments and to ensure uniformity during Q-angle, tibial torsion, and leg-length assessment. And the bony landmark was palpated directly on the skin; a soft skin pencil was used to mark the landmark before goniometer placement, ensuring that the marked reference point remained stable across the three repeated measurements.

**Figure 1:**
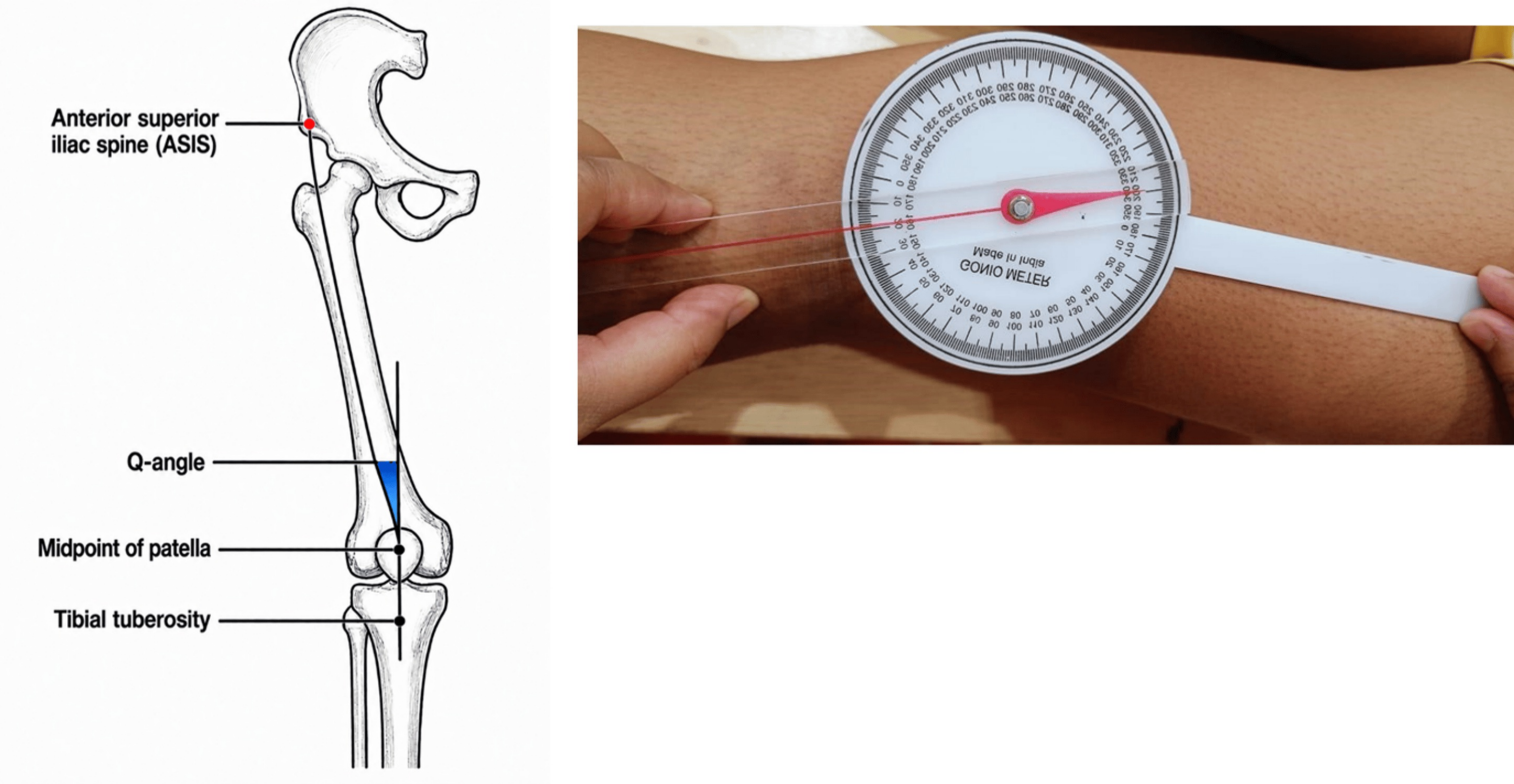
Goniometric assessment of quadriceps angle (Q-angle). Figure 1 shows the anatomical landmarks and goniometer alignment used for measurement of the quadriceps angle (Q-angle). Relevance: The Q-angle represents the resultant line of action of the quadriceps muscle on the patella during knee extension and is a key index of patellofemoral alignment [2,3]. Normal range: Published normative values are 10-14° in males and 13-17° in females [2,24]. Clinical implications: Values above the sex-specific upper limit are associated with lateral patellar tracking, patellofemoral pain syndrome, chondromalacia patellae, and a spectrum of overuse knee injuries [4-6]. ASIS: anterior superior iliac spine; Q-angle: quadriceps angle.

Q-angle: The Q-angle was measured using a universal goniometer following the method described by Lathinghouse and Trimble [23]. Participants were positioned supine with the knees in near-full extension. The supine, fully relaxed position was chosen deliberately because it isolates the static structural Q-angle from the dynamic contribution of active muscle pull and because it is the most widely used reference protocol in published normative studies, allowing direct comparison with prior literature [2,3,23,24]. The goniometer fulcrum was placed at the center of the patella, the stationary arm was aligned from the ASIS to the patellar midpoint, and the movable arm was directed from the patellar midpoint to the tibial tuberosity. The angle subtended between these two lines was recorded in degrees as the Q-angle (Figure 1).

**Figure 2:**
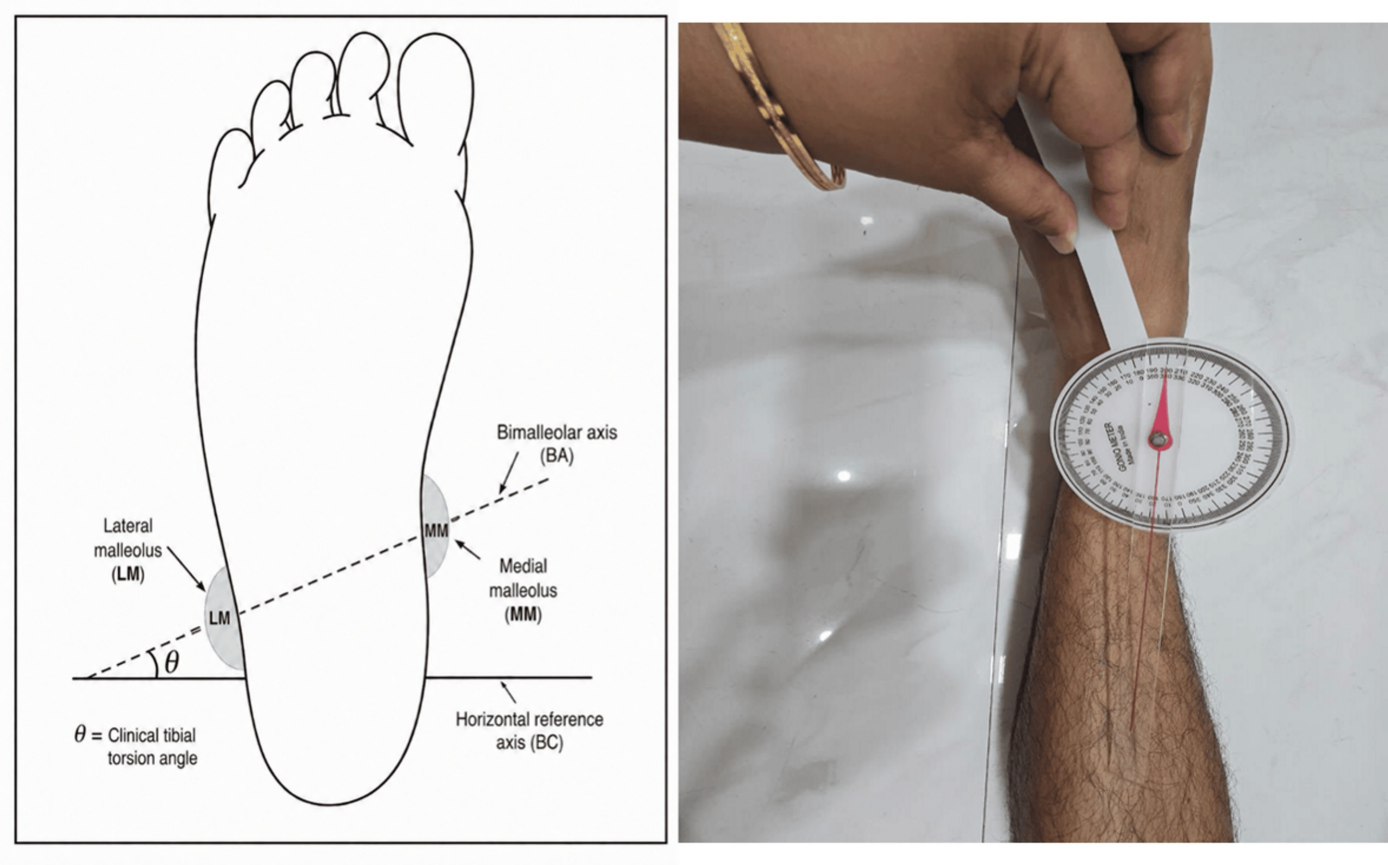
Goniometric measurement of tibial torsion. Figure 2 illustrates the clinical goniometric method used for tibial torsion assessment. Relevance: Tibial torsion represents the axial rotation of the tibia along its longitudinal axis and is a key determinant of foot progression angle, knee mechanics, and patellofemoral tracking. Normal range: The physiological range of external tibial torsion in adults is reported as approximately 15-30° in published goniometric and CT-based studies. Clinical implications: Excessive external tibial torsion laterally displaces the tibial tuberosity, increases the Q-angle, and predisposes to lateral patellar maltracking, patellofemoral pain, and overuse injury, whereas markedly reduced torsion is associated with in-toeing gait and altered knee mechanics. Q-angle: quadriceps angle.

Tibial torsion: Tibial torsion was assessed using a goniometric method [12] with participants in the supine position, knees extended, and femoral epicondyles aligned parallel to the examination table. The pivot of the goniometer was placed at the midpoint between the medial and lateral malleoli. The stationary opaque arm was placed along the bimalleolar axis from the medial to the lateral malleolus, while the movable transparent arm was oriented vertically and perpendicular to the examination table as the fixed reference axis (Figure 2). The angle between the bimalleolar axis and the vertical reference was read directly from the goniometer dial and recorded in degrees (Figure 2).

**Figure 3:**
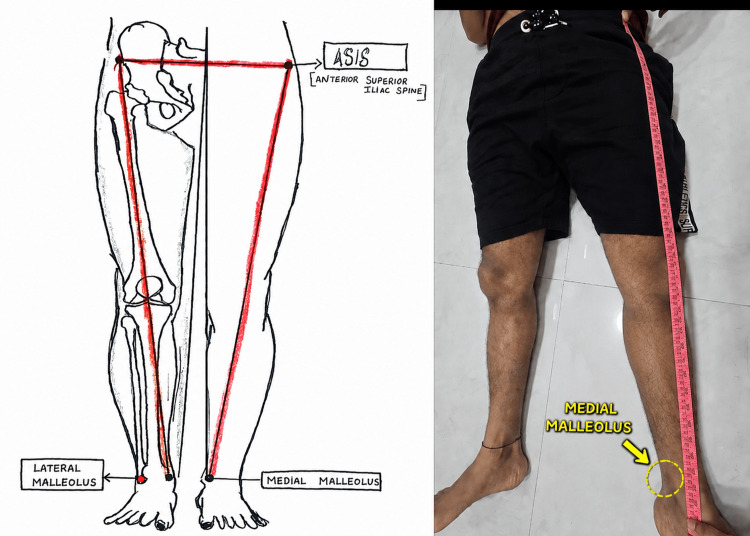
Total leg length measurement in the study participant. Figure 3 demonstrates the anatomical landmarks used for total leg length measurement. Note. Figure 3 depicts a linear anthropometric measurement (cm), not an angular parameter. Relevance: Total leg length is a structural anthropometric parameter measured to control for limb-length contribution to lower-limb biomechanics, since differences in leg length can independently influence pelvic tilt, gait symmetry, and the apparent Q-angle. Observed range: In the present Indian young-adult cohort, ASIS-to-malleolus leg length varied from 86 to 103 cm. Clinical implications: Leg-length asymmetry is associated with altered pelvic alignment and patellofemoral loading; in the present study, leg length was measured to confirm that any observed alignment differences between activity groups were not confounded by structural leg-length disparity. ASIS: anterior superior iliac spine; Q-angle: quadriceps angle.

Total leg length: Total leg length was measured in centimeters with the participant in the supine position by taking the linear distance from the ASIS to the medial and lateral malleoli using a calibrated non-stretchable measuring tape. The mean of the medial and lateral readings was used as the recorded leg length for analysis (Figure 3).

Measurement protocol and quality control

Each measurement, including Q-angle, tibial torsion, and stature and leg length, was repeated three times per participant, and the arithmetic mean of the three readings was used as the final recorded value to minimize random measurement error. Intra-observer reliability was supported procedurally by repeated measurements performed by the same examiner; however, formal statistical assessment using the intraclass correlation coefficient (ICC) was not performed and is acknowledged as a limitation. All measurements were performed by a single trained examiner throughout the study period to maintain uniformity in anatomical landmark identification and goniometer application technique. Raw data were compiled in Microsoft Excel (Microsoft Corporation, Redmond, Washington) spreadsheets with separate coding for sedentary and sports groups. Each participant was assigned a unique study code to maintain confidentiality, and all data were checked for completeness and consistency before statistical analysis. Outliers and missing values, if identified, were re-verified against the original records before analysis.

Statistical analysis

All data were analyzed using R (Version 4.3, Vienna, Austria) with the stats, coin, and boot packages. Continuous variables are presented as medians (IQR Q1-Q3) together with the observed range. The distribution of continuous variables was assessed using the Shapiro-Wilk test, which demonstrated significant departures from normality for Q-angle, tibial torsion, total leg length, weight, and BMI in one or both groups; the parametric Student’s t-test was therefore considered inappropriate. Between-group comparisons of continuous outcomes were performed using the two-tailed Mann-Whitney U test, and effect sizes were quantified using the rank-biserial correlation coefficient (r), interpreted as |r| < 0.1 negligible, 0.1-0.3 small, 0.3-0.5 medium, and >0.5 large. Ninety-five percent confidence intervals for the medians were obtained by bias-corrected and accelerated (BCa) bootstrap with 2,000 resamples. Sex-stratified between-group comparisons of every variable were performed as a pre-planned secondary analysis. Categorical variables were summarized as counts and percentages. Statistical significance was set at p < 0.05 using a two-tailed criterion.

## Results

Demographic and anthropometric characteristics

Age and Sex Distribution

The study participants were well-matched. Age distributions were identical between groups (median 22 years (IQR 20-24) in both groups; Mann-Whitney U = 20142.5, p = 0.901, r = 0.007, negligible effect). Sex was equally distributed, with 100 males and 100 females in each of the two cohorts (Table 1).

**Table 1:** Age distribution of sedentary and sports participants. Values expressed as median (IQR Q1-Q3). Between-group comparison was performed using the Mann-Whitney U test (U = 20142.5, p = 0.901, r = 0.007--negligible effect, not statistically significant). IQR: interquartile range.

Group	Median age (IQR) (years)	Range (years)	Mann-Whitney U
Sedentary	22 (20-24)	18-25	20142.5
Sports	22 (20-24)	18-25	

Anthropometric Profile: Height, Weight, and BMI

The two groups differed significantly in all three anthropometric parameters. Sedentary participants were taller and were also significantly heavier than those in the sports group (median 170 cm (IQR 165.5-176.0) vs. 168.75 cm (163.9-173.8); U = 22596, p = 0.025, r = 0.13, small effect). The BMI was higher in the sedentary group (median 23.81 kg/m² (21.2-26.9) vs. 22.98 kg/m² (20.0-25.2); U = 23901.5, p < 0.001, r = 0.20, small effect), consistent with the higher habitual energy expenditure expected in physically active young adults (Table 2).

**Table 2:** Comparison of anthropometric parameters--height (cm), weight (kg), and BMI (kg/m²)--between the sedentary (n = 200) and amateur athlete (n = 200) groups. Values expressed as median (IQR). Between-group comparisons were performed using the two-tailed Mann-Whitney U test; rank-biserial r was reported as the effect size. The statistical significance is at p < 0.05. BMI: body mass index; IQR: interquartile range.

Parameter	Sedentary median (IQR)	Sports median (IQR)	U (p; r)
Height (cm)	170 (165.5-176.0)	168.75 (163.9-173.8)	22596; p = 0.025; r = 0.13
Weight (kg)	68.5 (61.7-77.6)	64.3 (57.5-70.5)	25301.5; p < 0.001; r = 0.27
BMI (kg/m²)	23.81 (21.2-26.9)	22.98 (20.0-25.2)	23901.5; p < 0.001; r = 0.20

**Figure 4:**
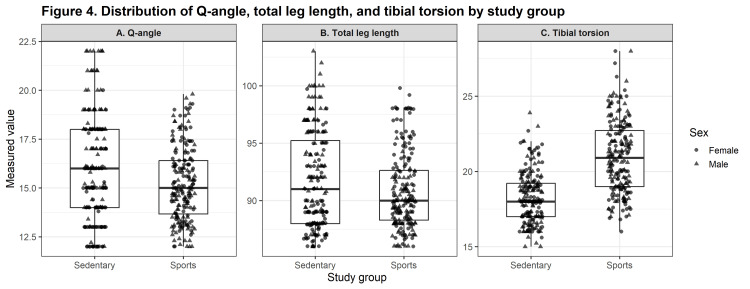
Box-and-whisker plots with jittered individual observations showing group-wise comparison of Q-angle, total leg length, and tibial torsion. Box-and-whisker plots with jittered individual observations showing group-wise comparison of Q-angle, total leg length, and tibial torsion between sedentary young adults and amateur athletes. Panel A shows the Q-angle distribution, panel B shows the total leg length distribution, and panel C shows the tibial torsion distribution. The box represents the IQR, the horizontal line within the box represents the median, and the whiskers indicate the observed spread of values. Individual observations are plotted as jittered points: circles denote female participants and triangles denote male participants. IQR: interquartile range; Q-angle: quadriceps angle.

Q-angle, total leg length, and tibial torsion (Figure 4).

Q-angle Distribution

The Q-angle across the entire cohort ranged from 12° to 22°; the sedentary group had a median Q-angle of 16° (IQR 14-18), whereas the sports group had a lower median Q-angle of 15° (IQR 13.7-16.4). The between-group difference was statistically significant on Mann-Whitney U testing (U = 23303, p = 0.004, r = 0.17, small effect), indicating that the sedentary group exhibited a wider Q-angle distribution. This pattern is illustrated graphically in Figure 4A and is consistent with diminished stabilizing musculature and altered patellofemoral mechanics in the sedentary cohort relative to the athletic cohort.

Total Leg Length Distribution

Total leg length ranged from 86 to 103 cm across the cohort. The sedentary group had a median leg length of 91 cm (IQR 88-95.2), and the sports group had a median of 90 cm (IQR 88.3-92.6). The between-group difference was not statistically significant (U = 21767.5, p = 0.126, r = 0.09, negligible effect), confirming that leg length behaved as a structural rather than activity-dependent anthropometric parameter in this cohort (Figure 4B).

Tibial Torsion Distribution

Tibial torsion values across the cohort ranged from 15° to 28°. The sedentary group had a median tibial torsion of 18° (IQR 17.0-19.2) with a BCa 95% CI of 18.0-18.3°, whereas the sports group had a markedly higher median tibial torsion of 20.9° (IQR 19.0-22.7) with a BCa 95% CI of 20.1-21.3°. The between-group difference was statistically significant on Mann-Whitney U testing (U = 7337, p < 0.001, r = −0.63, large effect) (Figure 4C).

The median difference between the groups was approximately 3°, representing a clinically meaningful upward shift of the tibial torsion distribution in the sports group relative to the sedentary group. The 95% CIs of the two groups did not overlap, strengthening the statistical interpretation of this finding. The interquartile ranges were narrow in both groups (2.2° and 3.7°, respectively), indicating intra-group homogeneity. Importantly, all observed values in both groups remained within the normal physiological range of 15-30°, as summarized in Table 3.

**Table 3:** Comparison of Q-angle (°), total leg length (cm), and tibial torsion (°) between the sedentary and amateur athlete groups. Values are expressed as medians (IQR Q1-Q3). Between-group comparisons were performed using the two-tailed Mann-Whitney U test. Effect size is reported as rank-biserial correlation (r). Reference Q-angle values: males 10-14°; females 13-17° [2,24]. Statistical significance was set at p < 0.05. IQR: interquartile range; Q-angle: quadriceps angle.

Parameter	Sedentary median (IQR)	Sports median (IQR)	U	Effect r	p value
Q-angle (°)	16 (14-18)	15 (13.7-16.4)	23303	0.17 (small)	0.004
Total leg length (cm)	91 (88-95.2)	90 (88.3-92.62)	21767.5	0.09 (negligible)	0.126
Tibial torsion (°)	18 (17-19.2)	20.9 (19-22.72)	7337	−0.63 (large)	<0.001

## Discussion

This study demonstrates that amateur athletes exhibit significantly greater tibial torsion than sedentary young adults (median 20.9° (IQR 19.0-22.7) vs. 18.0° (IQR 17.0-19.2); p < 0.001, r = −0.63, large effect), suggesting that regular sports participation correlates with measurable adaptations in tibial rotational alignment [25].

The study groups were well-matched for age, with both groups showing a median age of 22 years and an IQR of 20-24; p = 0.901, indicating that the results were not confounded by age-related developmental differences. Of the lower-limb biomechanical parameters assessed, Q-angle and tibial torsion showed statistically significant between-group differences. Anthropometric parameters also differed significantly between groups. Sedentary participants had higher BMIs (median 23.81 kg/m², IQR 21.2-26.9 vs. 22.98, IQR 20.0-25.2; p < 0.001, r = 0.20). The higher BMI among sedentary individuals is consistent with prior reports, indicating that physically active individuals exhibit a lower BMI due to enhanced energy expenditure and improved muscle tone maintenance [9,26].

The Q-angle was higher in sedentary than sports participants (median 16°, IQR 14-18 vs. 15°, IQR 13.7-16.4; p = 0.004, r = 0.17, small effect). The pre-planned sex-stratified analysis revealed a notable group-by-sex pattern: in the sedentary group, males had a higher median Q-angle than females (17.0° vs 15.0°), whereas in the sports group the relationship reversed (males 14.5° vs females 15.45°). This finding suggests that classical female-predominant Q-angle dimorphism [2,3] was attenuated by habitual activity in the present cohort and is consistent with the view that neuromuscular adaptation can modify sex-related alignment differences [3]. The overall Q-angle range was 12-22°, with group medians remaining close to published normative Q-angle values [2,24]. These findings align with previous studies reporting lower Q-angle values in physically active individuals and higher values in sedentary populations [27], attributed to improved muscle balance and joint stability in athletes; however, some studies have reported no significant difference, suggesting that training intensity and duration influence the degree of adaptation [24].

Mechanistically, lower Q-angle values in athletes can be explained by strengthening of the hip abductors, external rotators, and vastus medialis obliquus, which reduce dynamic knee valgus and improve patellar alignment, along with enhanced neuromuscular control that limits femoral internal rotation during weight-bearing [1]. In contrast, sedentary individuals tend to exhibit weaker stabilizing musculature and altered pelvic alignment, contributing to an increased Q-angle, while consistently higher values in females reflect anatomical factors such as a wider pelvis, increased femoral anteversion, and valgus knee orientation [2,3]. It is important to note that the present measurement was performed in the supine, fully relaxed position, which deliberately isolates the static structural Q-angle from active muscular contribution. The supine values, therefore, reflect the underlying skeletal alignment rather than dynamic neuromuscular control; standing and task-specific dynamic measurement, although introducing additional variability from active muscle pull and ground-reaction forces, would better reflect the functional sport-loading condition and represent a logical extension of the present work. Clinically, an elevated Q-angle increases lateral patellar tracking and predisposes individuals to patellofemoral pain, chondromalacia patellae, and overuse injuries [5], indicating greater biomechanical risk in sedentary individuals and more favorable patellofemoral mechanics in athletes, thereby supporting targeted strengthening interventions [1]. The total leg length did not differ significantly between groups (U = 21767.5, p = 0.126, r = 0.09), confirming that the observed differences in alignment parameters were functional rather than structural.

In contrast, the sports group demonstrated significantly greater tibial torsion than the sedentary group, with median values of 20.9° (IQR 19.0-22.7) vs. 18.0° (IQR 17.0-19.2), respectively (U = 7337, p < 0.001, r = −0.63), indicating adaptive variation rather than pathology [12,13]. Notably, gender differences were minimal in the sports group but present in the sedentary group, suggesting that physical activity may attenuate sex-based variation. This increase in tibial torsion in athletes is consistent with repetitive mechanical loading, which induces gradual rotational remodeling of the tibia, whereas sedentary individuals lack such stimuli.

Collectively, these findings demonstrate that regular sports participation correlates with measurable differences in tibial rotational alignment. While this observation is consistent with the hypothesis that mechanical loading may influence skeletal morphology [28], causation cannot be established from this cross-sectional design. Longitudinal studies are needed to determine whether sports participation drives these adaptations or whether individuals with specific alignment profiles are more likely to engage in athletic activity.

This study has several limitations that warrant consideration. First, the cross-sectional design precludes causal inference; although significant alignment differences were observed between sedentary and athletic groups, the present design cannot determine whether sports participation drives these adaptations or whether individuals with specific alignment profiles self-select into athletic activities. Second, convenience sampling from a single university population may limit the generalizability of the findings to non-student populations, rural communities, or other geographic regions. Third, goniometric measurement, while clinically feasible and ethically appropriate for healthy young adults, is less precise than radiographic or CT-based methods, which remain the imaging gold standard for both Q-angle and tibial torsion. No imaging-based validation against radiography or CT was performed in the present cohort because of the ethical constraints of exposing healthy young adults to ionizing radiation purely for research; published validation studies that have compared goniometric measurement with CT [10-12] support its clinical utility within its known measurement-error range. Fourth, each measurement was repeated three times per participant by a single trained examiner to minimize random error. Fifth, goniometric landmark identification, particularly of the ASIS, may be more challenging in participants with higher BMIs, which is a recognized constraint of skin-based goniometry compared with imaging-based measurement. Sixth, training intensity, training duration, sport type, femoral anteversion, pelvic tilt, and footwear characteristics were not assessed, although these variables may influence lower-limb alignment. Seventh, the study was conducted at a single center and did not evaluate functional outcomes such as knee pain, patellofemoral symptoms, or injury history; the cohort was deliberately restricted to participants without recent lower-extremity injury to establish a clean baseline. Future multicenter longitudinal studies that incorporate imaging-based validation, formal ICC-based inter- and intra-rater reliability, standing or task-specific dynamic measurement, and prospective correlation with knee pain and lower-extremity injury outcomes are warranted to strengthen these findings.

## Conclusions

This cross-sectional study of 400 young Indian adults demonstrates that amateur athletes exhibit a significantly lower Q-angle and greater tibial torsion expressed as median (IQR) compared with sedentary peers, with all values remaining within physiological ranges. These findings suggest that habitual sports participation correlates with distinct lower-limb rotational alignment profiles, likely reflecting biomechanical adaptations to repetitive loading. However, causation cannot be inferred from this observational design.

The elevated Q-angle observed in sedentary individuals may represent higher patellofemoral joint stress, while the increased tibial torsion observed in athletes appears to represent adaptive remodeling. These population-specific baseline values can inform pre-participation screening protocols in sports medicine and guide targeted interventions to optimize lower extremity biomechanics. Future longitudinal studies are warranted to establish temporal relationships and evaluate the functional outcomes associated with these alignment differences.
